# SCA: Search-Based Computing Hardware Architecture with Precision Scalable and Computation Reconfigurable Scheme

**DOI:** 10.3390/s22218545

**Published:** 2022-11-06

**Authors:** Liang Chang, Xin Zhao, Jun Zhou

**Affiliations:** School of Information and Communication Engineering, University of Electronic Science and Technology of China, Chengdu 611731, China

**Keywords:** look-up table, search-based computing, precision scalable, reconfigurable, computational utilization

## Abstract

Deep neural networks have been deployed in various hardware accelerators, such as graph process units (GPUs), field-program gate arrays (FPGAs), and application specific integrated circuit (ASIC) chips. Normally, a huge amount of computation is required in the inference process, creating significant logic resource overheads. In addition, frequent data accessions between off-chip memory and hardware accelerators create bottlenecks, leading to decline in hardware efficiency. Many solutions have been proposed to reduce hardware overhead and data movements. For example, specific lookup-table (LUT)-based hardware architecture can be used to mitigate computing operation demands. However, typical LUT-based accelerators are affected by computational precision limitation and poor scalability issues. In this paper, we propose a search-based computing scheme based on an LUT solution, which improves computation efficiency by replacing traditional multiplication with a search operation. In addition, the proposed scheme supports different precision multiple-bit widths to meet the needs of different DNN-based applications. We design a reconfigurable computing strategy, which can efficiently adapt to the convolution of different kernel sizes to improve hardware scalability. We implement a search-based architecture, namely SCA, which adopts an on-chip storage mechanism, thus greatly reducing interactions with off-chip memory and alleviating bandwidth pressure. Based on experimental evaluation, the proposed SCA architecture can achieve 92%, 96% and 98% computational utilization for computational precision of 4 bit, 8 bit and 16 bit, respectively. Compared with state-of-the-art LUT-based architecture, the efficiency can be improved four-fold.

## 1. Introduction

Computer-vision-based applications, such as image classification and object detection, are widely used in industrial detection, automatic driving, and robot brains. With the development of deep learning theory, more and more artificial intelligent (AI) vision applications based on convolution neural networks have achieved significant improvements in the corresponding metrics compared with traditional methods, including improved accuracy and mean average precision (mAP) [1,2,3,4,5,6,7,8]. In recent years, AI computer vision applications have progressively penetrated into daily life in areas such as face recognition, smart homes and automatic driving. However, with the iteration and optimization of algorithms, the network scale becomes larger with a more complex structure. Using traditional hardware, such as CPUs and GPUs, it is difficult to achieve satisfactory results in terms of speed and power, which inevitably creates new challenges for the design of efficient domain-specific architectures (DSAs) [9,10].

To improve the efficiency of hardware computation, a large number of neural-network-specific acceleration methods and corresponding accelerators have been proposed, including the optimization of computation and data access [11,12,13]. Traditional multiplication and addition (MAC) operations account for the majority of computation in neural network inference, which creates excessive logic resource overheads and power consumption in the process of hardware implementation. To solve this problem, hardware based on fast convolution algorithms, such as Winograd and img2col, were proposed [14,15,16]. The authors of [14] proposed a super-resolution accelerator based on the Winograd algorithm, which accelerated convolution by reducing the number of multiplications, and ultimately achieved a real-time, super-resolution reconstruction speed of 120 fps. However, as the convolution kernel and tile size increase, the cost of addition and transform must be considered. In particular, the aforementioned methods still entail expensive multiplication. To eliminate multiplication, ref. [17] proposed an AddrNet-based accelerator, iMAD, to replace the computationally intensive multiplication in the convolution layer with lightweight addition and subtraction, resulting in an energy efficiency improvement of 3.55×. Using sparsity, ref. [18] proposed a bit-level hardware architecture SAC, which can complete the partial sum operation by operating the essential bits in the parameters, thus replacing the traditional MAC operation without any loss of accuracy. The authors of [19] proposed a Bitlet accelerator design based on a bit interleaving scheme which was able to achieve up to 15× higher speed-up over the fixed-point accelerator, Laconic [20]. Based on a pruning scheme, ref. [21] proposed a hardware runtime pruning methodology BitX and achieved 4.82× speed-up over the original non-pruned DNN.

Look-up table (LUT)-based computing is another method which has been used to replace traditional MAC operation. The multiplication results are stored in the LUT in advance and each computing operation only needs to find the corresponding result from the LUT. Because there is no logic computation, the computing speed can be greatly improved. The corresponding accelerators include LACC, DRISA and SCOPE [22,23,24,25,26]. For example, LACC effectively decreases the LUT size by enabling LUT-based vector multiplication in DRAM with a 6.3× efficiency improvement [22]. The authors of [24] adopted stochastic computing to simplify the complex multiplication. However, in the process of neural network inference, frequent data interaction with off-chip memory will lead to a decline in computing performance and an increase in hardware power. Therefore, ref. [27] adopted a layer fusion mechanism to reduce the off-chip DRAM bandwidth by 92%. In [28], a block convolution method was proposed, which divides the feature map into several independent small feature maps, thus avoiding the transmission of middle-layer feature map data to the off-chip memory. The authors of [29,30] proposed a tile-based selective SR chip, SRNPU, which can reduce communication bandwidth with external memory by 78.8%, enabling efficient caching of intermediate feature maps with a short reuse distance.

Although LUT-based computing avoids multiplication, implementation of LUT leads to a large amount of memory overhead. With increase in computational precision to support larger bit width, the memory resource overhead increases exponentially and the chip area overhead becomes unacceptable. In addition, how to efficiently support the convolution of different kernel sizes is also an important issue. In light of the issues described above, we propose an efficient search-based computing architecture, termed SCA, which not only reduces memory overhead, but also improves the utilization and versatility of computation. In addition, the on-chip structure greatly reduces data access with off-chip memory.

The main contributions of this paper are as follows:

- We propose an efficient search-based computing scheme, which greatly reduces the memory overhead compared with the traditional LUT method by optimizing the LUT and adopting an efficient encoding technique. In addition, the designed search-based computing macro (SC macro) supports a variety of computational bit widths, which can adapt to network models with different precision requirements and improve the versatility of the architecture.
- We propose a reconfigurable computing strategy. Based on the designed SC macro, it can dynamically support the convolution of different kernel sizes with high computational utilization.
- We implement a search-based on-chip architecture SCA, which can greatly reduce interactions with off-chip memory, alleviate the bandwidth pressure, and improve computing efficiency.

The remainder of this paper is organized as follows: Section 2 introduces the basic principles of lookup table computation and low bit neural networks and then analyzes existing problems. Section 3 discusses the design of the search-based computing macro, considering precision scalable and computation reconfigurable techniques. Section 4 discusses the design of the search-based on-chip architecture. Section 5 presents a detailed analysis of the experimental results. Section 6 summarizes the paper.

## 2. Preliminary and Motivation

This section discusses the background to the current research and LUT-based computation. In addition, we describe current developments in the quantization of neural networks. Finally, we summarize the challenges faced and our motivation for undertaking this research.

### 2.1. Computing with LUT Memory

**Figure 1:**
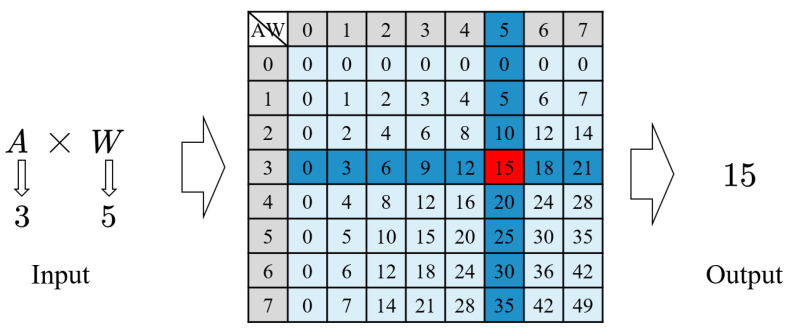
Multiplication process based on LUT. The multiplication result is obtained by searching in the LUT according to the input data pair. A and W represent activation and weight, respectively.

As a widely used component in digital circuit systems, LUTs can perform various logic operations, data storage and other functions [31,32,33,34]. For LUT-based computation, the LUT stores the results of all multiplications in advance. During each multiplication, the results are retrieved in the LUT according to a pair of data inputs and then multiplication is completed, as shown in Figure 1. Compared with the traditional MAC operation, the LUT-based computation method replaces logic operations with search operations, completing multiplication and other operations in a short period. Therefore, cascading LUTs can readily improve computational parallelism. However, with increase in computational bit width, exponentially increased memory resources for results storage are required. The specific storage entry requirements are represented by Equation (1), where AW and BW represent the bit widths of the input data and *P* represents the computational parallelism. For example, for a multiplication with a bit width of 8 bits and a parallelism of 10, 655,360 storage entries are required and the resulting memory overhead will overwhelm the advantages of the LUT.

(1) $$\#\;of\;entries=P\times \left(2^{AW}\right)\times \left(2^{BW}\right)$$

### 2.2. Neural Network under Quantization

**Figure 2:**
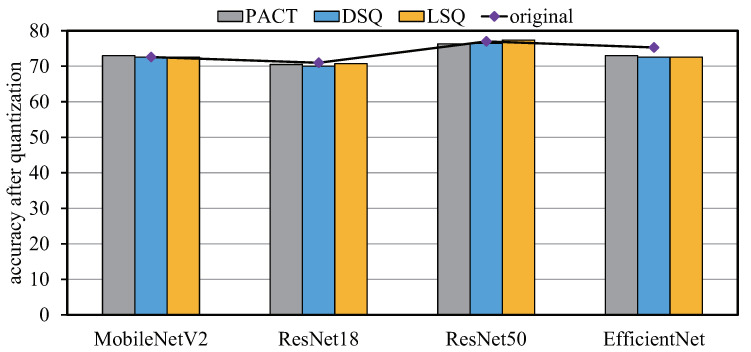
Model quantization comparison using different methods at 4-bit precision. Original line indicates the accuracy without quantization.

Typically, the inference and training of neural network algorithmsemploy floating-point precision to ensure a higher level of accuracy. However, high precision computation requires large computing logic resources and more cycles for hardware implementation. To reduce computation and memory resources, an increasing number of designs based on model quantization have been proposed which quantize the model to 8 bits or even lower to 4 bits [35,36,37]. For example, ref. [35] proposed a parametric clipping activation technology PACT, which used the activation clipping parameter α optimized during training to find the right quantization scale, with negligible accuracy loss (only 0.3% degradation in ResNet50). As shown in Figure 2, through a series of experiments, neural network models were quantized to 4 bits with almost no accuracy loss, where the quantized model greatly reduced the hardware overhead and relieved the bandwidth pressure.

### 2.3. Challenges and Explorations

LUT-based computation faces several challenges for widespread deployment. Firstly, a significant amount of memory resources are needed to store the multiplication results. In particular for high-precision computing, the storage entries grow exponentially, which significantly undermines the advantages of LUT computing. In addition, improving the generality of LUT computation and adapting to convolution with different bit widths and kernel sizes are important issues requiring consideration. Furthermore, the bandwidth pressure caused by frequent data accesses with off-chip memory represents a significant obstacle to improved hardware performance.

Based on our observations, we analyzed the LUT-based computation principle and its effects on the hardware overhead. We propose an SCA architecture to reduce data storage and data movements. We explore the 4-bit LUT as the baseline to optimize memory overhead based on the quantization trend of the network. Then, higher bit precision computation is supported. With a reconfigurable scheme, the proposed SCA architecture supports the convolution operation with different kernel sizes. Moreover, we develop an on-chip architecture to reduce frequent data accesses to improve the performance of the SCA architecture.

## 3. Efficient Search-Based Computing Scheme

In this section, we discuss the LUT optimization method. Based on the optimal LUT computing engine, we design a search-based computing macro (SC macro). Applying the reconfigurable feature of LUT-based computing, we are able to develop processing units with various bit widths and kernel sizes.

### 3.1. LUT Optimization

**Figure 3:**
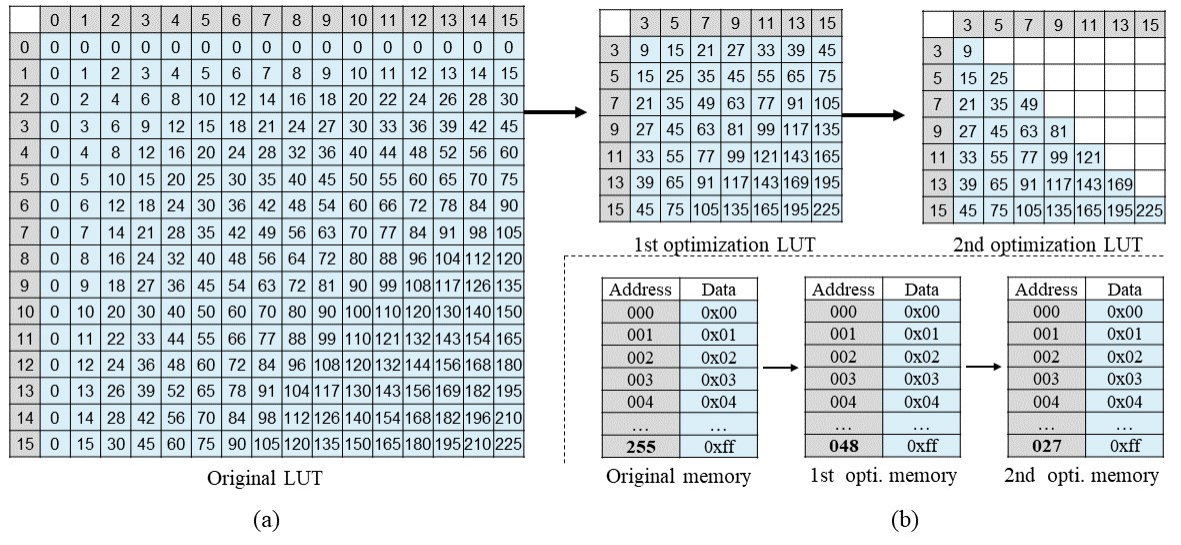
Optimization of 4-bit LUT. (**a**) 4-bit LUT optimization. 1st optimization decreases the even part of the input data pair; 2nd optimization decreases the same output by taking the diagonal as the boundary. (**b**) 4-bit LUT memory. After two optimizations, the number of storage entries are decreased from 256 to 28.

Figure 3a indicates the 4-bit LUT to support multiplication with all possible input combinations. According to Equation (1), 256 multiplication results need to be stored in advance; thus, 256 storage entries are required to correspond to the original memory, as shown in the lower-left of Figure 3b. In the original 4-bit LUT, there are many repeating data entries which increase the memory resource overhead. Based on this observation, we can reduce the number of storage entries using two-step optimization. In the first optimization step, we remove the even part of the LUT input data pair as described in [38]. The multiplication of even data and of other data (odd or even) includes two cases: ❶ It is decomposed into the multiplication of two odd numbers, and the multiplication result is then shifted to obtain the final result. The result of the odd data multiplication can be obtained directly by searching in the LUT; thus, the multiplication can be simplified into a search and a shift operation. ❷ If there are even data with a power of two in the input data pair, the final result can be achieved by shifting the other data directly without searching in the LUT. According to the above discussion, the even part of the LUT input can be removed, so that the storage entries are reduced from 256 to 49, as shown in Figure 3 for the first optimization LUT and corresponding memory. In the second optimization step, we can also remove the stored results on the symmetrical side of the diagonal in LUT, as shown in the second optimization LUT of Figure 3a. The diagonal line is a dividing line that matches the computation results on both sides; the storage entries can be reduced from 49 to 28, as shown in the lower-right of Figure 3b.

Through the above two-step optimization, the 4-bit LUT storage entries are reduced from 256 to 28, reducing the storage entries by 89%, thus alleviating the pressure on the memory overhead.

### 3.2. Search-Based Computing Macro

**Figure 4:**
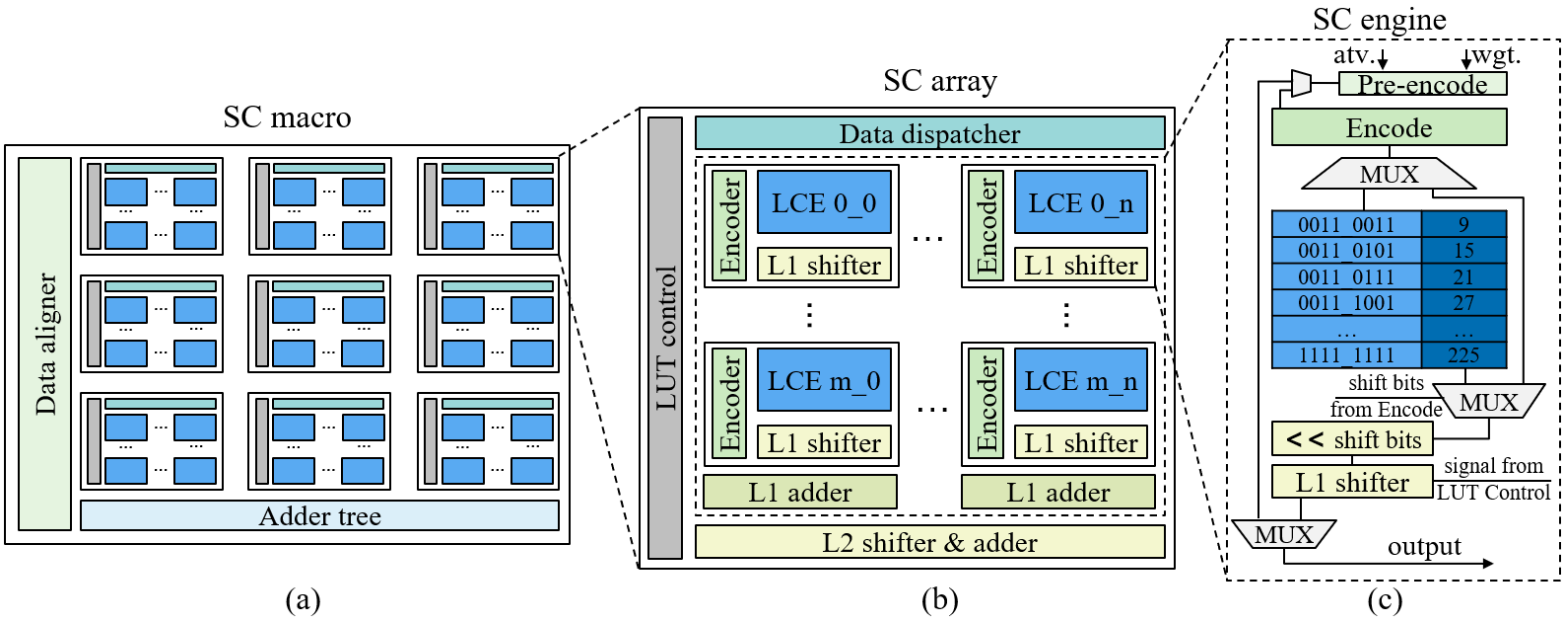
Structure of SC (search-based computing) macro. (**a**) SC macro, which contains 9 SC arrays. (**b**) SC array, which includes 16 SC engines. LCE indicates the LUT-based computing engine. (**c**) SC engine, the basic computing unit.

Based on the optimization method, we develop a search-based computing (SC) macro design, as shown in Figure 4a, including a data aligner, nine SC arrays and an adder tree. The SC macro supports different computing modes, such as different bit widths and kernel sizes, using the data aligner. The data aligner can distribute the input data to different SC arrays. Each SC array completes one or more multiplications and the adder tree performs the summation and accumulation of the partial results output by SC arrays. The structure of each SC array is shown in Figure 4b and consists of a data dispatcher, 16 SC engines (basic computing unit based on LUT) with a shape of 4 × 4, two-level shifters and adders and a control unit. The data dispatcher allocates the input data to the specific SC engine according to the different computing modes. In addition, the two-level shifters and adders are in different switching states according to the different computational bit widths.

The basic computing unit in the SC macro is shown in Figure 4c. The input data pair, activation (atv.) and weight (wgt.) with a bit width of 4 bits, are first pre-encoded to determine whether they can be output directly or require search and shift operations before output. In the encoder, the input data is further analyzed to determine whether the data needs search, shift, even decomposition and other operations. Then, the length of the required shift is analyzed and, finally, the encoding sequence is output. If the input data does not need to search, it is sent directly to the shifter to shift according to the shift length. In the case of 4-bit computing bit width, no further shift is required, so the L1 (level 1) shifter is skipped.

### 3.3. Efficient Encoding Scheme

The specific implementation of the encoder in the SC engine is shown in Algorithm 1, including pre-encoding and encoding. According to the input data pair with a width of 4 bits, a sequence with a length of 8 bits, and the corresponding shift length, are finally output. Pre-encoding is used to judge whether the input data contains 0 or 1. If it contains 0, it directly outputs 0 and ends the current computing; If it contains 1, it directly outputs another data value and skips the subsequent search and shift operations.

In the encoding process, we first judge the parity of the input data pair, and further assess whether it is a power of two for even data. In sum, there are five cases: ❶ odd and odd, ❷ odd and even, ❸ odd and even (power of two), ❹ even and even, ❺ even (power of two) and even (or both even data values are powers of two). In case ❶, the input data pairs are directly spliced into an output sequence without shift operation. In case ❷, even data are decomposed to obtain an odd data value and the corresponding shift length, and then output after splicing two odd data values. For example, when the input is 7 and 12, 12 is decomposed into 3 × 4, then 7 and 3 are spliced to obtain an output sequence of 0111_ 0011 and the shift length is 010 (4 is the second power of 2.). The output sequence is sent to the LUT for searching to obtain the output of 21 and the shift length of two is sent to the shifter to obtain the final output of 84. In case ❸, the even data of the power of two are decomposed into shift length, skip LUT, and output directly after shifting. In case ❹, the two even data are decomposed and then spliced to obtain the output sequence and the corresponding shift length. In case ❺, the even data of one of the powers of two is decomposed and then shifted and output. In summary, all cases of 4-bit multiplication are considered.
**Algorithm 1** Pseudo code of encoding scheme**Input:** 
activation and weight pair: a[3:0], w[3:0]**Output:** 
output data or search sequence: o[7:0]; shift bits: s[2:0]1:**def pre-encoding**(a, w)2:       **if**(a == 0 or w == 0)3:              **return** o=0; s=None;4:       **else if**(a ==1 or w ==1)5:              **return** o=(a == 1) ? w : a; s=None;6:**def encoding**(a, w)7:       data, sel = **judge**(a, b)8:       **case**(sel)9:              odd, odd :                                              ▹ case 110:                   **return** o={data[odd], data[odd+1]}, s=None;11:             odd, even :                                               ▹ case 212:                   **return** o={data[odd],**decomp**(data[even])[0]},13:                            s=**decomp**(data[even])[1];14:             odd, even_x :                                              ▹ case 315:                   **return** o=data[odd], s=**decomp**(data[even_x])[1];16:             even, even :                                           ▹ case 417:                   **return** o={**decomp**(data[even])[0], **decomp**(data[even+1])[0]},18:                            s=**decomp**(data[even])[1]+**decomp**(data[even+1])[1];19:             default :                                              ▹ case 520:                   **return** o=data[even], s=**decomp**(data[even_x])[1];21:**void main**(a, w)22:       **pre-encoding**(a, w)23:       **encoding**(a, w)1Function **judge** to determine the parity of input data.2Function **decomp** to decompose the data to multiple odd data.

### 3.4. Precision Extension Strategy

**Figure 5:**
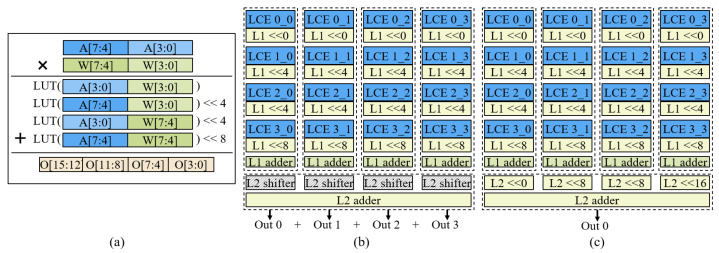
Precision scalable strategy. (**a**) Logic process for 8-bit multiplication using LUT. (**b**) Case for 8-bit multiplication, which outputs 4 data for SC array at a time. (**c**) Case for 16-bit multiplication, which outputs 1 data value for the SC array at a time.

The SC macro supports multiplication with a bit width of 4 bits, which can adapt to most neural network applications. However, for some applications that require high computational precision, such as super-resolution, it is necessary to expand the bit width to support the computation for higher precision. Based on this, we propose a precision extension strategy. Figure 5a shows the computing process of multiplication with a bit width of 8 bits. First, we decompose each 8-bit input into two data values with a bit width of 4 bits. Then, four 4-bit multiplication results are obtained by combining the computations of the 4-bit data. Finally, according to the position of the 4-bit input data in the original 8-bit data, these are shifted and added, respectively, to obtain the result of the 8-bit multiplication. Mapping to the specific SC array is shown in Figure 5b. For an SC array, each column contains four SC engines; with the L1 shifters and adder, an 8-bit multiplication can be completed. The SC array contains four columns, which can complete a total of four 8-bit multiplications. The L2 shifters are skipped, the four 8-bit multiplication results in the L2 adder are added, and finally the sum of the four 8-bit multiplications is output.

Similarly, we expand the bit width from 8-bit to 16-bit using the same method above, which requires a two-step decomposition. In the first decomposition, the 16-bit multiplication is decomposed into four 8-bit multiplications. In the second decomposition, each 8-bit multiplication is decomposed into four 4-bit multiplications. In this way, a 16-bit multiplication can be decomposed into sixteen 4-bit multiplications. As shown in Figure 5c, in the SC array, each column completes one 8-bit multiplication, and four columns complete four 8-bit multiplications in total. Then, the 8-bit result is shifted through the L2 shifter, and, finally, a 16-bit multiplication result is output after addition in the L2 adder.

To highlight the efficiency of our proposed strategy, the method proposed in this paper is compared with the traditional method. For 8-bit multiplication, the traditional method requires a LUT with a bit width of eight bits. According to Equation (1), a total of 65,536 storage entries are required. In this paper, only four optimized LUTs with a bit width of four bits are needed, with a total of 112 storage entries, thereby reducing the number of storage entries by 585 times. For 16-bit multiplication, the traditional method requires 4.3 × 10${}^{9}$ (4,294,967,296) storage entries, while our method only needs 448 storage entries, a total reduction of 9.6 × 10${}^{6}$ (9,586,980) times, to store the entries. It can be seen that our proposed strategy can efficiently support high-precision multiplication, which compares favorably with traditional methods.

### 3.5. Reconfigurable Computing Strategy

**Figure 6:**
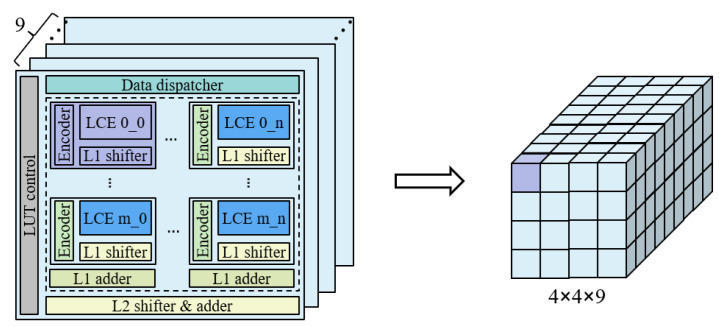
SC macro structure simplification. Each SC engine is represented by a small cube and the whole macro is converted to a large cube of dimensions 4 × 4 × 9.

In Section 3.4, we discuss the efficient implementation of multi-precision computation. After solving this problem, we face a new challenge —how to effectively support the convolution of different kernel sizes. In other words, for the convolution of different kernel sizes, how can it be ensured that the SC macro always maintains high computational utilization? For the convenience of analysis, we simplify the structure of the SC macro, as shown in Figure 6. Each SC engine is mapped into a small cube, which can perform a 4-bit multiplication; the whole macro is mapped, and, finally, a large cube of dimensions 4×4×9 is obtained.

#### 3.5.1. Data Mapping for 3×3 Convolution

**Figure 7:**
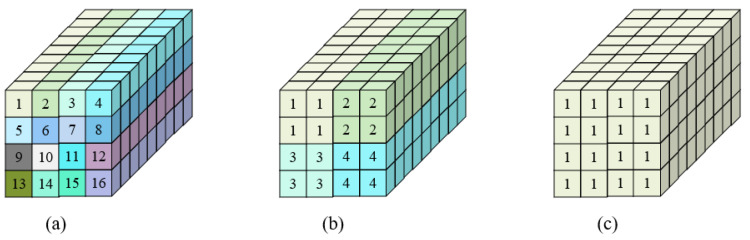
Data-mapping for 3×3 convolution. (**a**) Case for 4-bit, which completes 16 convolutions in total at a time with utilization 100%. (**b**) Case for 8-bit, which completes 4 convolutions in total at a time with utilization 100%. (**c**) Case for 16-bit, which completes 1 convolution in total at a time with utilization 100%.

First, we discuss data mapping for the 3×3 convolution; a 3×3 convolution requires nine multiplications. As shown in Figure 7a, when the bit width is set as 4-bit, each small cube can complete a multiplication and the depth of the entire big cube is nine, which can complete exactly nine multiplications, corresponding to the computation of a 3×3 convolution. Further, the width and height of the big cube are both four; thus, a total of sixteen 3×3 convolutions can be completed. The specific data arrangement is shown in the figure; small cubes with the same color correspond to the same convolution. When the bit width is 4-bit, all the small cubes are used and the computational utilization of the SC macro is 100%.

For a 3×3 convolution with a bit width of 8 bits, since one multiplication is achieved by each of the four small cubes, a cube of dimensions 2×2×9 can complete a 3×3 convolution, as shown in Figure 7b. For the whole big cube, a total of four 3×3 convolutions can be achieved and the computational utilization rate is 100%. The bit width is further extended to 16 bits; sixteen small cubes are required for one multiplication, so one SC macro can complete one 3×3 convolution with 100% computational utilization, as shown in Figure 7c.

#### 3.5.2. Data Mapping for 1 × 1 Convolution

**Figure 8:**
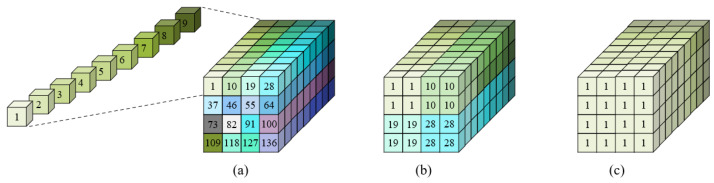
Data-mapping for 1 × 1 convolution. (**a**) Case for 4-bit, which completes 144 convolutions in total at a time with 100% utilization and each cube finishes one 1 × 1 convolution. (**b**) Case for 8-bit, which completes 36 convolutions in total at a time with utilization 100%. (**c**) Case for 16-bit, which completes 9 convolutions in total at a time with utilization 100%.

For a 1 × 1 convolution, based on the original 3×3 convolution, the number of convolutions supported is expanded by nine times. As shown in Figure 8a, for 4-bit computation, the computation block which can complete one 3×3 convolution before can complete nine 1 × 1 convolutions now, so that the whole SC macro can complete 144 convolutions with size 1 × 1 (i.e., 4 × 4 × 9). For a 1 × 1 convolution with 8 bits, each of the four small cubes completes a 1 × 1 convolution; the depth of the big cube is nine, so the cubes with size 2 × 2 × 9 can support nine 1 × 1 convolutions. For the whole SC macro, a total of 36 1 × 1 convolutions are obtained (i.e., 2 × 2 × 9), as shown in Figure 8b. Similarly, for 16-bit computing, the whole macro can support nine convolutions with size 1 × 1 (i.e., 1 × 1 × 9), as shown in Figure 8c. For the above three bit widths, the computational utilization is 100%.

#### 3.5.3. Data-Mapping for 5 × 5 Convolution

**Figure 9:**
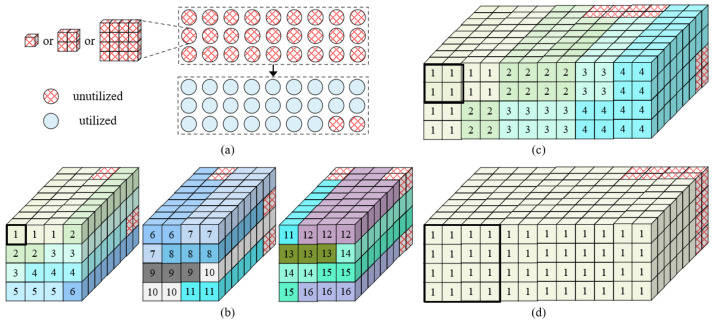
Data-mapping for 5 × 5 convolution. (**a**) Utilization analysis. (**b**) Case for 4-bit, 3 macros compute 16 convolutions with utilization 92.6%. (**c**) Case for 8-bit, 3 macros compute 4 convolutions with utilization 92.6%. (**d**) Case for 16-bit, 3 macros compute 1 convolution with utilization 92.6%.

For a 5 × 5 convolution, 25 multiplications are required. As shown in Figure 9a, since the depth of the SC macro is nine, to improve its utilization as much as possible, a 5 × 5 convolution can be achieved by the computation block with a size of 1 × 9 × 3. Each basic computing unit represents cubes of different sizes according to the different computing bit widths. For example, when the bit width is 8 bits, each computing unit is composed of cubes with the size of 2 × 2. According to the figure, for each 5 × 5 convolution, two basic computing units are not used, so the computational utilization rate is 92.6%.

When the bit width is 4 bits, a 5 × 5 convolution requires cubes with a size of 1 × 1 × 9 × 3; we take three SC macros as a whole, and a total of sixteen 5 × 5 convolutions can be completed (i.e., (4 × 4 × 9 × 3)/(1 × 1 × 9 × 3)) maintaining a computational utilization rate of 92.6%, as shown in Figure 9b. When the bit width is 8 bits, a convolution needs cubes with a size of 2 × 2 × 9 × 3. Then every three SC macros can complete four 5 × 5 convolutions (i.e., (4 × 4 × 9 × 3)/(2 × 2 × 9 × 3)); the computational utilization rate is 92.6%, as shown in Figure 9c. Similarly, as shown in Figure 9d, for a computation with a bit width of 16 bits, every three SC macros can complete one 5 × 5 convolution (i.e., (4 × 4 × 9 × 3)/(4 × 4 × 9 × 3)); the computational utilization rate is also 92.6%.

Based on the above discussion, the developed SC macro can support the convolution of different bit widths and of different kernel sizes without introducing an additional logic overhead and has high computational utilization.

## 4. SCA: Search-Based Computing Architecture

This section provide the architecture based on the optimized LUT and SC macro. In addition, the on-chip storage mechanism is discussed.

### 4.1. Architecture Overview

**Figure 10:**
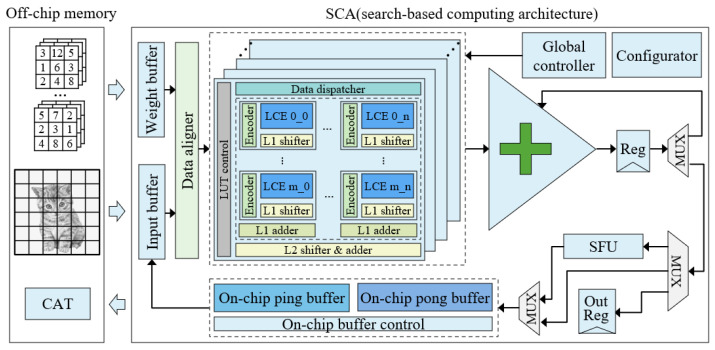
Search-based computing architecture (SCA). Off-chip memory stores input images, weight, and output results. SCA completes the calculation of the whole network.

Based on the developed SC macro, we implemented a search-based computing architecture, namely SCA, as shown in Figure 10. It mainly comprises an input buffer, weight buffer, SC macro group, special function unit (SFU), on-chip buffer, global controller, configuration unit and other components. The input buffer and weight buffer temporarily store feature map data and weight data, respectively. As the core computing component, the SC macro affects the convolution of different bit widths and kernel sizes. The SFU completes activation, pooling and other non-linear operations. The on-chip buffer stores the output feature map data of the middle layers. The global controller controls the inference of the whole network. The configuration unit is used to initialize the parameters of the whole network, including the computational bit width, the number of network layers, the number of channels in each layer, the size of the convolution kernel and other parameter information.

The dataflow of the SCA architecture includes several steps. Firstly, the SCA is initialized and the configuration unit obtains the configuration information. Secondly, the weight buffer loads the weight data and the input buffer loads the input feature map data. Thirdly, the global controller reads the configuration information of the first layer from the configuration unit and controls the whole architecture to complete the computation of the first layer. Next, the global controller switches the state, obtains the configuration information of the second layer and repeats the above operations until the computation of the last layer is completed. The final output result is sent to the output register (Out Reg).

### 4.2. On-Chip Storage Mechanism

As mentioned in Section 2.3, frequent data accesses with the off-chip memory represent a significant challenge, leading to a decline in performance and an increase in power consumption. Based on the SCA architecture, we provide an on-chip storage structure. In some neural networks, only feature maps of the first layer and the last layer are required to transfer with the off-chip memory. The data for the middle layers can be stored entirely in the on-chip buffer, which greatly reduces data movements between the on-chip buffer and the off-chip memory.

**Figure 11:**
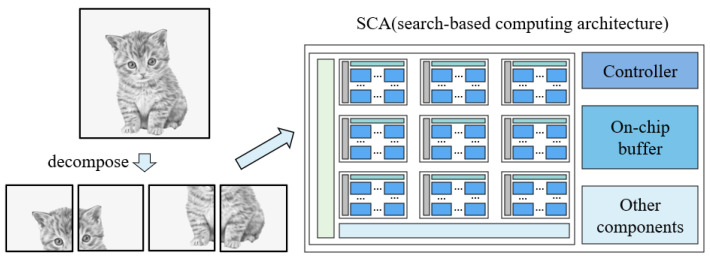
Feature map decomposition.

For some applications, such as super-resolution, the output feature map data of the middle layer is larger than the capacity of the on-chip buffer and cannot be stored in the on-chip buffer. In this case, we develop a feature map decomposition strategy, which decomposes the original large input feature map into multiple small feature maps and then sends them to the SCA architecture for processing in turn, finally splicing them for output, as shown in Figure 11. To minimize the impact of the decrease in accuracy caused by feature map decomposition, we also decompose the training set during training.

## 5. Experiment and Evaluation

In this section, we describe the experimental setup and performance analyses of the proposed SCA architecture. In addition, comparison with other investigations is discussed. Finally, we present our analyses on the SCA architecture and discuss future work.

### 5.1. Experiment Setup

To evaluate our approach, Verilog HDL was used to complete the modeling of each component, based on the TSMC 28 nm library, to determine parameters such as area and power consumption. On the basis of the above, we built an in-house simulator to cascade the components to implement the simulation and testing of the whole architecture. In the practical evaluation, we chose AlexNet, VGG16 and ResNet50 as the test networks. Specifically, we evaluated the following cases: SC 4b, SC 8b and SC 16b, which represent the computing bit widths of the SCA architecture of 4 bits, 8 bits and 16 bits, respectively.

### 5.2. Area and Power

**Figure 12:**
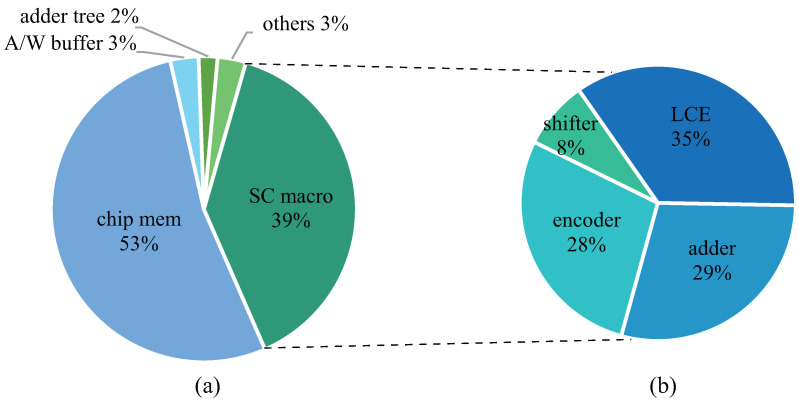
Area breakdown. (**a**) Area breakdown of SCA architecture. (**b**) Area breakdown of SC macro.

Figure 12a shows the area breakdown of the SCA architecture in which the on-chip buffer occupies more than half of the area of the whole architecture. In addition, the area proportion for the SC macro ranks second, while the other components, such as the adder tree and controller, only occupy a small part of the area. It can be seen that the memory occupies the main part of the SCA architecture. There are two main reasons for this. First, the traditional multiplication is implemented by the LUT based on search, so that the computation logic is replaced by storage. Secondly, an on-chip computing structure is adopted, so a large piece of memory is added to store the feature map data. Further, we divide the SC macro to analyze its area composition. As shown in Figure 12b, the LCE occupies the largest area; that is, the LUT has the largest area overhead in the SC macro. In addition, the encoder only occupies a small part of the area in the SC engine, which shows that the implementation of the encoder is efficient.

**Figure 13:**
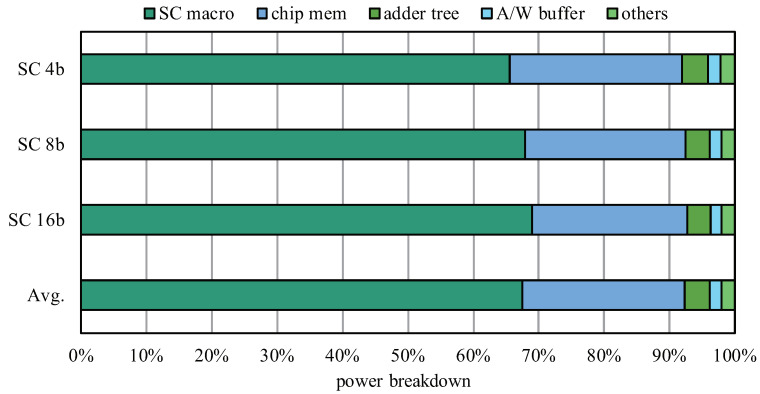
Power breakdown for different computational bit widths.

Figure 13 shows the power breakdown of the SCA architecture. Since the computation power is different under variable computational bit widths, we evaluate them accordingly. It can be seen from the figure that, under different bit widths, the SC macro occupies the main power, because the parallelism of the SC macro is 144 (4 × 4 × 9); that is, there are a maximum of 144 SC engines working at the same time, so the power consumption it brings occupies a major role. In addition, by comparing the power proportion of each component under different bit widths, it can be observed that the main difference comes from the SC macro and that the proportion of the SC 16b is the largest. The main reason is that, when the bit width is 16 bits, the two-level shifters and adders are in a working state, so larger power consumption will occur.

### 5.3. Macro Utilization

As discussed in Section 3.5, for different computational bit widths (i.e., 4 bits, 8 bits, and 16 bits), when the convolution size is 1 × 1 or 3 × 3, the computational utilization rate can reach 100%. A 5 × 5 convolution can also achieve a high computational utilization of 92.6%. However, the above situation is only the optimal computational utilization. In practical neural network applications, there may be different degrees of computational utilization decline due to the size of the convolution and the number of channels in each layer of the network.

**Figure 14:**
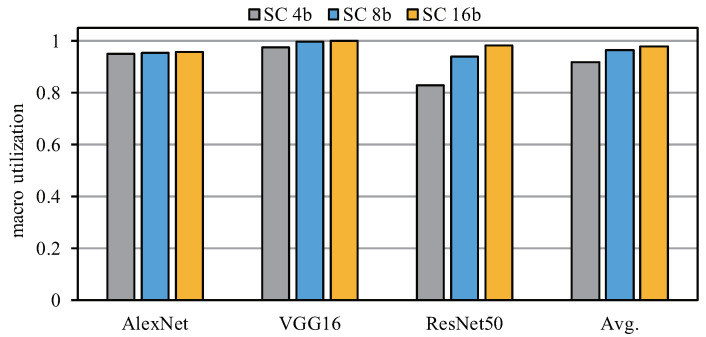
Utilization for different bit widths with different networks.

Figure 14 shows the actual computational utilization of the SCA architecture in different networks. When the bit width gradually increases from 4 bits to 16 bits, the computational utilization also increases. The main reason is that the computing parallelism of a higher bit width is lower (that is, the maximum number of computations that the SC macro can support), which causes the degree of unused SC macro computing resources to be lower. In addition, for the VGG16 model, the computational utilization tends to be 100% under different bit widths. By analyzing the network structure, we can observe that the whole network only contains 1 × 1 and 3 × 3 convolutions and the number of channels in most network layers can be divided by SC macro parallelism, thus ensuring high utilization. By averaging the computational utilization of different networks, it can be observed that the computational utilization is greater than 90% and the utilization of SC 8b and SC 16b is higher than 95%. Therefore, the proposed method has high utilization in different networks and the SCA architecture shows good robustness.

**Figure 15:**
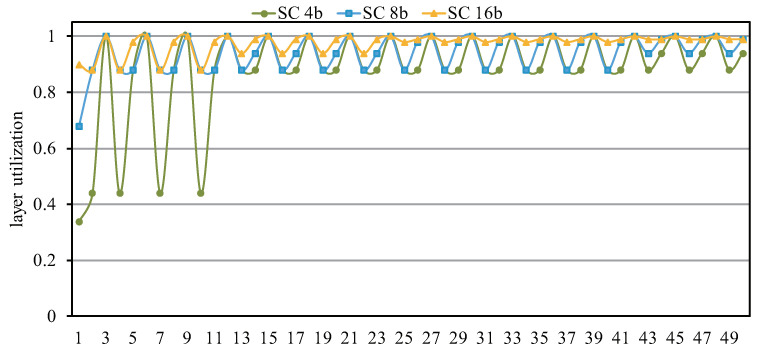
Layer utilization for different bit widths with ResNet50.

We consider ResNet50 as an example to analyze the computational utilization of each layer. As shown in Figure 15, the curve where SC 16 is located is at the highest position and has the highest computational utilization. In addition, the decrease in SC 4b computational utilization is mainly caused by the low computing utilization of the previous layers, which has two main aspects. On the one hand, the first layer includes a 7 × 7 convolution, though we use two 5 × 5 convolutions splicing to complete it; since the input channel of the first layer is three and the parallelism of SC 4b is 16, there is a waste of 62% of computing resources. In addition, the inherent waste of computing resources in the 5 × 5 convolution also leads to low utilization of the first layer of computing. On the other hand, the other layers with low utilization are 1 × 1 convolutions and the number of channels is low while the convolution parallelism is too large, resulting in a waste of more computing resources.

### 5.4. Performance

**Figure 16:**
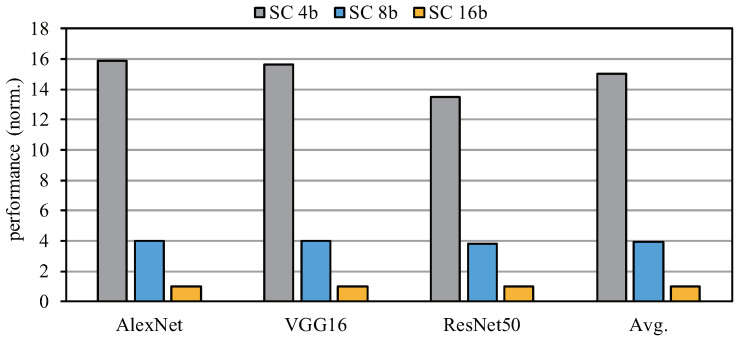
Performance comparison for different bit widths with different networks.

Figure 16 shows a performance comparison of the SCA architecture. Compared with SC 16b, SC 4b can achieve a maximum performance acceleration of 15 times. It can be seen that SCA has higher performance under low bit width. In addition, it can be seen from Figure 14 that AlexNet and ResNet50 have lower computing utilization than VGG16 under SC 4b. Therefore, the experimental results in Figure 16 show that their performance is also relatively lower. Through the average performance comparison, we can see that SCA has more significant advantages in low-bit-width computation.

### 5.5. Efficiency

Compared with the traditional un-optimized LUT, the SC engine proposed by us requires less storage entries to store the multiplication results, so it has higher area efficiency. To demonstrate the efficiency of the SC engine, we replace the SC engine with a traditional un-optimized LUT and embed it into the SCA architecture for testing. Since the SCA architecture supports three different bit widths: 4 bit, 8 bit and 16 bit, there are six test cases.

**Figure 17:**
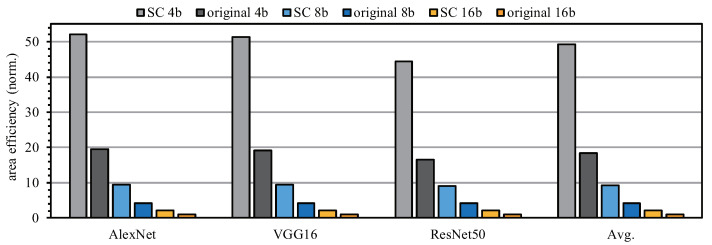
Area efficiency comparison for different bit widths with different networks.

As shown in Figure 17, for each computational bit width, our proposed SC-engine-based architecture has higher area efficiency than the original LUT-based method in different test networks. In AlexNet and VGG16, the area efficiency of the SC 4b is more than 50 times higher that of the original 16b. Taking the average of all the network test results under different bit widths, the area efficiency of the SC engine is at least twice that of the method based on the original LUT. It can be seen that our method results in a significant efficiency improvement compared with the original method.

### 5.6. Comparison with Conventional Logic-Based Computing Hardware

To compare the advantages of our proposed LUT-based SCA architecture over conventional logic-based computing hardware in terms of power and area efficiency, the SCA was compared with a CPU and a GPU. The configurations of the CPU and the GPU were Core i5-7200U and RTX2080Ti, respectively, and all experimental data were based on a VGG16 network. As shown in Table 1, the SCA had the lowest power, which was only 0.019 mW, much less than the CPU and GPU. Correspondingly, the area overhead was 0.08 mm^2^, also much less than the CPU and GPU. In addition, the SCA had the highest area efficiency—482 times and 15 times higher than the CPU and GPU, respectively. With respect to latency, our proposed SCA had a latency 22 times higher than the CPU. However, it was slightly lower than the GPU, the main reason being that our hardware scale was much smaller than the GPU. When we used the same computing area overhead as the GPU, that is, the area was consistently set to 754 mm^2^, our architecture SCA power consumption was only 79% of the GPU, but the computing latency was improved to 1260 times that of the GPU.

### 5.7. Comparison with Other LUT-Based Studies

**Figure 18:**
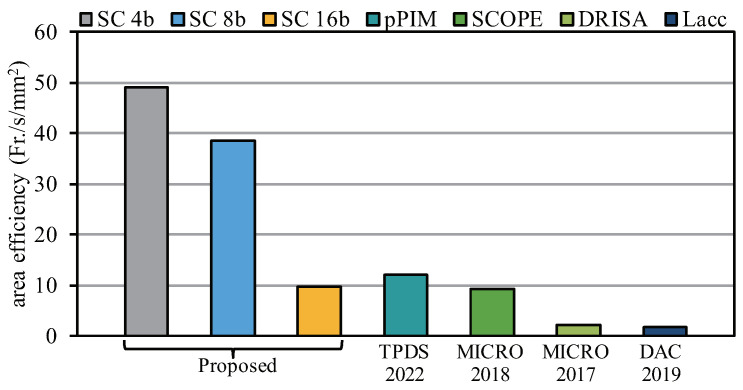
Area efficiency comparison with other works.

To further compare the efficiency of our proposed architecture, we compared the SCA architecture with other LUT-based investigations. Figure 18 shows the area efficiency for the different architectures. We were able to achieve a maximum efficiency improvement of 28 times at a precision of 4 bits. In addition, compared with state-of-the-art LUT-based methods, we were still able to achieve a four-fold increase in efficiency. When the computational bit width was 16 bits, the computational parallelism was greatly reduced for the SCA architecture, resulting in a reduction in the area efficiency, which was slightly lower than that of pPIM. However, in other cases, the efficiency of the SCA architecture was much higher than that found in other studies, indicating the high efficiency of our architecture.

### 5.8. Discussion

#### 5.8.1. Trade-Off Analysis

**Figure 19:**
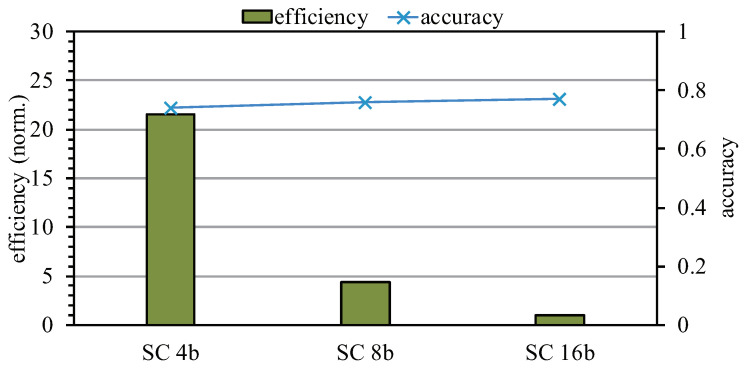
Trade-off analysis of SCA architecture.

From the above analysis, we can see that a lower computational bit width can achieve higher computational efficiency. However, as the computational bit width decreases, the accuracy of the network will also decrease. As shown in Figure 19, taking ResNet50 as an example, we can see that the efficiency of SC 4b was about 21 times higher than that of SC 16b, but the accuracy hardly decreased. Therefore, on the whole, for most applications that are not constrained by a strict computational bit width, using a low bit width will result in improved hardware efficiency.

#### 5.8.2. Hardware Overhead Analysis

In Section 3, we analyze computation schemes with different computing precision. Due to the redundancy of the design, when the computing precision is different, the computing resources required for actual computation are different; that is, the computing overhead is different. When the computing precision is 4-bit, the minimum computing precision of our LUT can support 4-bit; so we do not need any shifter which is redundant in this pattern and the computing resource overhead is 8.8%. When the computing precision is set to 8-bit, we do not need the second-level shifter (L2 shifter) and the computing resource overhead is 2.5%. When the computing precision is set to 16-bit, we need all the shifters, and the corresponding computing hardware is nearly zero. In sum, when the computing precision is maximum, the computing overhead is minimum, and the hardware utilization is maximum.

#### 5.8.3. Scalability Analysis

The SCA architecture proposed in this paper adopts a computing method based on search and the computation is flexible. Therefore, according to the requirements of practical application scenarios, it is easy to expand in terms of computing bit width, convolution size and computing parallelism. If we need a higher computational precision, we can easily expand it. For example, for 32-bit computing, we can use two SC macros. In addition, for convolution computation, it can be extended to support convolution of other sizes. For example, for 7×7 convolution, a computing array of size 6×9 can be used which has 91% computational utilization. Moreover, for scenarios with high computing parallelism requirements, it is also convenient to cascade multiple SC macros to improve the degree of parallelism.

## 6. Conclusions

In this paper, we proposed a search-based computing architecture, SCA, which was shown to eliminate traditional multiplication and greatly improve computational efficiency. In addition, the architecture supported convolution with multiple bit widths and kernel sizes, which greatly improved the versatility and robustness of the architecture. Based on the experimental evaluation, the SCA architecture demonstrated high computational utilization—the computing efficiency was four times higher than that for state-of-the-art LUT-based methods. In future work, based on the scalability of the SCA architecture, we aim to improve the architecture further to support more applications with higher efficiency.

## Figures and Tables

**Table 1:** Comparison with Conventional Logic-based Computing Hardware.

Hardware	Power (W)	Area (mm^2^)	Latency (ms)	Area Efficiency (GOPS/mm^2^)
CPU	7.47	149	343.1	0.4
GPU	225	754	2.1	12.7
SCA (proposed)	0.019	0.08	15.8	192

## Data Availability

Not applicable.
